# Personalised Prescription of Scalable High Intensity Interval Training to Inactive Female Adults of Different Ages

**DOI:** 10.1371/journal.pone.0148702

**Published:** 2016-02-05

**Authors:** Jacqueline L. Mair, Alan M. Nevill, Giuseppe De Vito, Colin A. Boreham

**Affiliations:** 1 UCD Institute for Sport & Health, Newstead, University College Dublin, Belfield, Dublin, Co Dublin, Ireland; 2 School of Sport, Performing Arts and Leisure, University of Wolverhampton, Wolverhampton, West Midlands, United Kingdom; 3 School of Sport, Ulster University, Shore Road, Newtownabbey, Co Antrim, United Kingdom; Victoria University, AUSTRALIA

## Abstract

Stepping is a convenient form of scalable high-intensity interval training (HIIT) that may lead to health benefits. However, the accurate personalised prescription of stepping is hampered by a lack of evidence on optimal stepping cadences and step heights for various populations. This study examined the acute physiological responses to stepping exercise at various heights and cadences in young (n = 14) and middle-aged (n = 14) females in order to develop an equation that facilitates prescription of stepping at targeted intensities. Participants completed a step test protocol consisting of randomised three-minute bouts at different step cadences (80, 90, 100, 110 steps·min^-1^) and step heights (17, 25, 30, 34 cm). Aerobic demand and heart rate values were measured throughout. Resting metabolic rate was measured in order to develop female specific metabolic equivalents (METs) for stepping. Results revealed significant differences between age groups for METs and heart rate reserve, and within-group differences for METs, heart rate, and metabolic cost, at different step heights and cadences. At a given step height and cadence, middle-aged females were required to work at an intensity on average 1.9 ± 0.26 METs greater than the younger females. A prescriptive equation was developed to assess energy cost in METs using multilevel regression analysis with factors of step height, step cadence and age. Considering recent evidence supporting accumulated bouts of HIIT exercise for health benefits, this equation, which allows HIIT to be personally prescribed to inactive and sedentary women, has potential impact as a public health exercise prescription tool.

## Introduction

The health and fitness benefits of stair climbing have been established in both epidemiological and intervention studies. Habitual stair climbing has been associated with reduced BMI [1], reduced risk of premature mortality [2–4] and stroke [5] as well as increased longevity [6]. Additionally, short-term intervention trials have found stair climbing to be an effective, time efficient strategy for improvements in aerobic fitness [7–9] and certain health parameters, such as lipid profiles [8, 10]. For example, Kennedy and colleagues [9] report that 6 minutes of stair climbing per day resulted in comparable improvements in aerobic fitness as walking for 45 minutes per day. It is likely that such health outcomes are a result of the accumulation of short bouts of vigorous intensity stair climbing exercise (8.8 METs; [11]), which is substantiated by investigations linking short duration, high intensity interval training (HIIT) with health benefits [12–14].

Despite the desirable health and fitness outcomes associated with stair climbing, the activity does present a number of barriers and is not suitable for some populations. For example, older individuals have a higher risk of falling while climbing stairs [15], resulting in a higher incidence of such falls [16]. Furthermore, many people may not have access to multiple flights of stairs during their everyday routine and of those who do, several report a stair climbing threshold of no more than four flights of stairs before they will choose to take the lift [17]. Thus, a similar exercise modality, which overcomes the barriers to stair climbing while replicating its benefits, may be of value for public health interventions. Bench stepping is an analogous activity to stair climbing which may fulfil these criteria.

Bench stepping requires an individual to ascend and descend a single step of specified height by completing a four-step cycle (two steps upwards onto the bench, and two steps downwards off the bench). It has been classified as vigorous intensity exercise of approximately 8.5 METs [11], which is similar to stair climbing at a moderate to brisk pace [8, 18, 19]. It is currently unclear, however, what step height and step cadence combination is required to achieve this intensity, especially in populations of different ages. Additionally, the MET intensity allocated to this activity is based on a standardised resting VO_2_ of 3.5ml·kg^-1^·min^-1^ [20] a reference value that is insensitive to differences in age, gender and body mass index (BMI; [21]).

It is important to establish an equivalency between stepping and stair climbing so that the latter (with its proven health benefits [1–10]) may be replicated with bench stepping. This is particularly important if stepping is to be promoted in populations of different ages, as it is well established that physical work capacity reduces with age [22, 23]. Furthermore, previous work from our group has helped to characterise the neuromuscular demands of bench stepping [24], as well as improvements in cardiorespiratory fitness, and mobility arising from short-term bench step training [25, 26]. However, there remains a need to further refine the prescription of step exercise especially considering its potential as a scalable HIIT model that may be promoted on a public health level.

This study, therefore, aims to describe and compare the physiological responses to bench stepping exercise in young and middle-aged females, in order to develop an equation that will allow the prescription of step exercise (using step height and step cadence) at a target intensity known to enhance health and fitness in a given population. The chosen step heights for this study are commonly found in public and residential settings (17 cm; or 34 cm if two are taken at a time) and compare with the heights from a standard, commercially available aerobic bench (25 cm and 30 cm). Our choice of step height therefore facilitates the prescription of stepping exercise based on an individual’s everyday environment and promotes activity that may be easily incorporated into the daily routine.

## Methods

The study was approved by the ethics committee of University College Dublin and was performed in accordance with the ethical standards laid down in the 1964 Declaration of Helsinki. Fourteen young (aged 19–30 years) and 14 middle-aged (aged 50–63 years) females were recruited for the study through advertisements in local area and university newsletters (Table 1). Participants were provided with a full explanation of the study and written informed consent was obtained prior to testing. Inclusion criteria were: females aged 18–30 years and 50–64 years who are physically inactive (as defined by a failure to meet recommended activity levels [27]), but medically stable [28]. Exclusion criteria included cigarette smoking, anabolic steroid ingestion, a history of cardiovascular disease, diabetes (type I or type II), hypertension, or any other metabolic disease or illness requiring the ingestion of medications that affect carbohydrate or lipid metabolism.

**Table 1:** Participant characteristics.

	Young	Middle-Aged	t	Cohen’s *d*
**Age (years)**	23.4 ± 2.74	56.6 ± 3.99		
**Stature (m)**	1.68 ± 0.66	1.65 ± 0.04	1.78	0.67
**Body Mass (kg)**	64.8 ± 9.65	64.7 ± 6.57	0.01	0.01
**BMI (kg·m**^**2**^**)**	23.0 ± 3.35	23.9 ± 2.47	-0.87	0.33
**Leg Length (cm)**	87.4 ± 3.19	86.0 ± 2.71	1.24	0.47
**RMR (kcal)**	1363.1 ± 288.12	1041.2 ± 153.57	3.64*	1.37
**VO**_**2**_**rest (mL·kg**^**-1**^**·min**^**-1**^**)**	3.08 ± 0.59	2.36 ± 0.36	3.90*	1.47

*significant difference between-groups (p = 0.001). RMR = resting metabolic rate. BMI = body mass index. Values are means ± SD, n = 14 in each group, df = 26.

### Research Design

The experimental design required participants to report to the exercise physiology laboratory on three occasions, separated by a minimum of seven days. During the first session participants were measured for anthropometric variables and resting metabolic rate (RMR), and on the second and third sessions, participants completed a step test protocol (described below).

### Anthropometric variables

Stature was determined with a stadiometer (Holtain Ltd, Pembs, UK) to the nearest 0.1cm, and body mass was measured on a digital scale (Seca, Hamburg, Germany) to the nearest 0.1kg with the participant wearing minimal clothing and no shoes. BMI was calculated using the equation *body mass/height*^*2*^. Leg length was measured to the nearest 0.1cm with the subject in a supine position taking a line from the anterior superior iliac spine to the medial malleolus using a standard tape measure.

### Resting Metabolic Rate

RMR measurements were carried out following a recommended protocol [29]. Participants were instructed to abstain from food and beverages (except water) for a minimum of 12 hours and to avoid strenuous exercise for 24 hours prior to testing. In addition, all participants travelled by car or public transport on the day of testing so as not to raise metabolism significantly above resting levels. RMR was measured using a Quark b^2^ indirect calorimeter (Cosmed Srl, Rome, Italy) following standard manufacturer calibration procedures, and resting HR was measured using a telemetric heart rate monitor (Polar Electro Oy, Kempele, Finland). On arrival at the laboratory, participants rested quietly in a supine position for 20 minutes in a temperature-controlled room. Measurement commenced immediately following the rest period, using a facemask protocol, and respiratory quotient values were monitored throughout the test to ensure accurate gas measurements. Data were collected at 30-second intervals for a period of 15 minutes and analysed offline for steady state.

### Dietary Control Measures

All participants completed a two-day nutritional diary on the days leading up to the second testing session. This was then replicated prior to the third testing session to reduce variability in metabolic measures that may have resulted from a change in diet. Participants were instructed to abstain from food and beverages three hours before testing, not to drink coffee or beverages containing caffeine for at least eight hours and to avoid intense exercise for 48 hours prior to testing.

### Stepping Exercise Test

**Fig 1:**
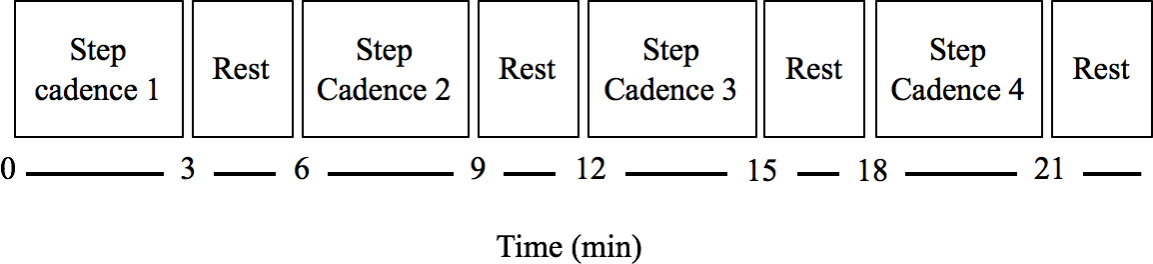
Stepping test protocol.

The stepping test protocol (Fig 1) consisted of randomised three-minute stepping bouts at four different cadences (80, 90, 100, 110 steps·min^-1^) interspersed by three-minutes of rest to allow heart rate to return to within 10 beats of baseline values. This protocol was repeated using four different step heights (17, 25, 30, 34 cm), two completed during session two and two completed during session three, also selected in a random order. Participants therefore completed a total of 16 three-minute stepping bouts. The stepping cycle consisted of two steps up onto the bench to full extension of the legs and two steps back down, maintaining the same leading leg throughout. Therefore a cadence of 100 steps·min^-1^ equated to 25 step cycles. Step cadence was regulated using a pre-selected music track with the required tempo. All participants performed a familiarisation trial at each treatment combination of step height and step cadence prior to the commencement of testing on both days. A handrail was provided as an added safety precaution in the event that the participant should lose balance during the test, but care was taken to ensure that the handrail was not used to aid the stepping cycle.

Throughout the stepping protocol, the participant wore a face-mask (Hans Rudolf Inc, Shawnee, KS, USA) connected to an indirect calorimeter (Cosmed Srl, Rome, Italy) for breath-by-breath measurements of gas exchange, and a telemetric heart rate monitor (Polar Electro Oy, Kempele, Finland) for continuous measurement of heart rate (HR). The gas analyser was calibrated with known concentrations of O_2_ and CO_2_ and the turbine was calibrated with a three-litre syringe prior to every testing session. Baseline resting values of VO_2_ and HR were collected for two minutes immediately preceding commencement of the stepping protocol.

### Data Analysis

Resting metabolic rate was calculated from 10 minutes of steady state data (defined as variation in the volume of oxygen consumed of no more than 10% [30]) and in all cases, the initial five minutes of test data were discarded. Heart rate reserve (HRr) was calculated [31] using maximum HR determined from a female specific equation [32], and mean resting HR obtained during the RMR test. Mean values for VO_2_ (ml·kg^-1^·min^-1^), HR (beats·min^-1^), HRr (%) and metabolic cost (Kcal·min^-1^) were calculated from the final 30 s of filtered data at each step height/step cadence combination during the stepping test, and METs were calculated by dividing mean VO_2_ by the corresponding individual resting VO_2_ value obtained during the RMR test.

### Statistical Analysis

Statistical procedures were carried out using SPSS 20 (IBM Corporation, New York, USA). Sample size was established by performing an *a priori* power calculation based on previously published data [33]. Using a two-tail multiple regression model with a significance level of 5%, an effect size of 0.82 (derived from an R^2^ value of 0.45), and 3 predictor variables, this analysis suggested a minimum of 14 participants per group would achieve a power of 85%.

Normal distributions were inspected using the Shapiro-Wilk test and normal Q-Q plots prior to analysis. An independent samples t-test was used to test between-group differences for anthropometric variables and RMR values and effect size values were calculated using Cohen’s *d*, whereby 0.2 is considered a small effect, 0.5 a medium effect, and 0.8 a large effect [34]. A three-way ANOVA with repeated measures was used to analyse dependant variables (METs, HR, HRr, metabolic cost), with the three factors being a between-subjects factor of ‘age group’ (the young vs. middle-aged) and two within-subjects factors of ‘step height’ and ‘step cadence’. *Post-hoc* pairwise comparisons with Bonferroni correction were used to identify where any detected differences lay and effect sizes were calculated using partial eta squared (η_p_^2^) where 0.01 is a small effect, 0.06 is a medium effect, and 0.14 is a large effect [34]. A multilevel regression analysis [35] on the combined data (young and middle-aged groups) was performed using Multilevel Models Project (MLn) software in order to establish a prediction equation for the purpose of prescribing stepping exercise at a target intensity (METs). The two-level hierarchical regression model used to predict METS incorporated three fixed-factor predictor variables; step height, step cadence and age, with participants or individuals, assumed to be a random sample, being the level two units, and with the subjects’ repeated measurements, being the level one units. Standard errors obtained from maximum-likelihood estimation procedures were used to establish the significance of the model. Statistical significance was accepted as *P*<0.05.

## Results

There were no differences between groups for any measured anthropometric variables. RMR was significantly lower in the middle-aged females compared with the young (Table 1). Furthermore, resting VO_2_ was 12% and 33% lower than the standard resting value (equivalent to 1 MET) of 3.5ml·kg^-1^·min^-1^ in the young and middle-aged group, respectively.

### Between-Group Analysis

**Fig 2:**
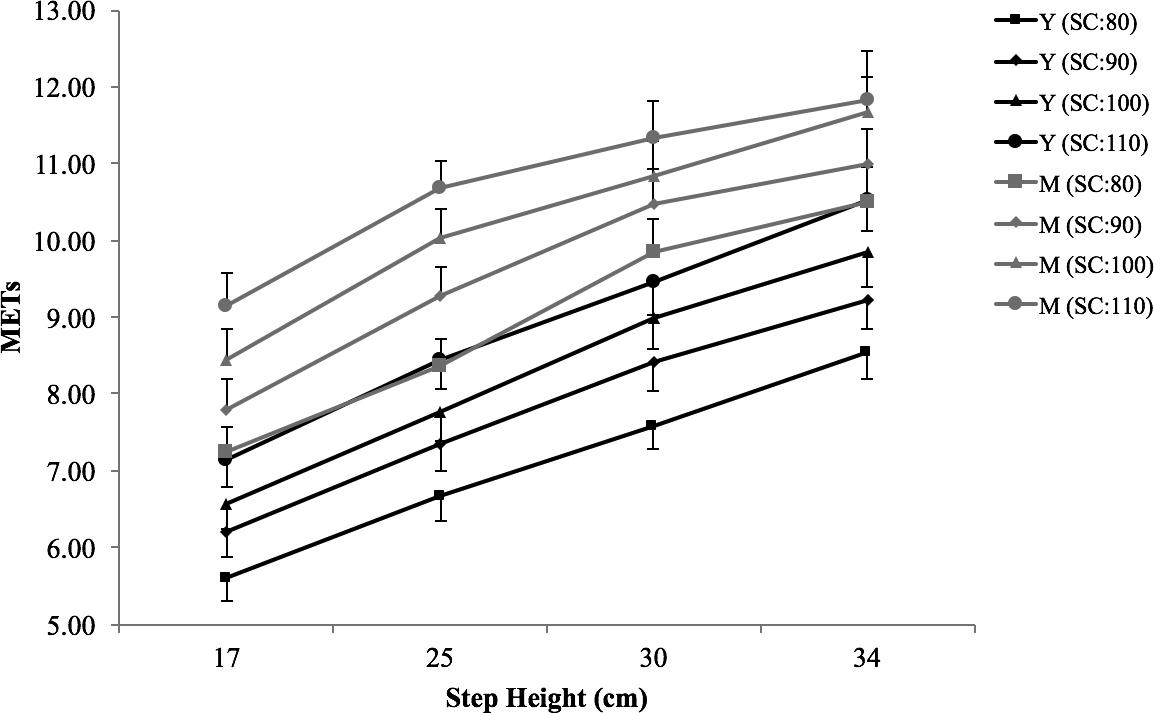
METs across step height and step cadence combinations. Values are means ± SE; Y = young females; M = middle-aged females; SC = step cadence.

The repeated measures ANOVA revealed significant between-group main effects across step height and step cadence combinations for METs (*P*<0.01, η_p_^2^ = .350; Fig 2) and %HRr (*P*<0.01, η_p_^2^ = .366), but there were no observable differences for HR (*P* = 0.84, η_p_^2^ = .002), or metabolic cost (*P* = 0.81, η_p_^2^ = .002). At a given step height and cadence, the middle-aged females were required to work at an intensity on average 1.9 ± 0.26 METs greater than the younger females.

### Within-Group Analysis

Within-group comparisons (Table 2) revealed a significant main effect for step height across all dependent variables; METs (*P*<0.001, η_p_^2^ = .904), HR (*P*<0.001, η_p_^2^ = .864), %HRr (*P*<0.001, η_p_^2^ = .864) and metabolic cost (*P*<0.001, η_p_^2^ = .932). Similarly, a significant main effect for step cadence was observed for all dependent variables; METs (*P*<0.001, η_p_^2^ = .893), HR (*P*<0.001, η_p_^2^ = .940), %HRr (*P*<0.001, η_p_^2^ = .942) and metabolic cost (*P*<0.001, η_p_^2^ = .924).

**Table 2:** Physiological responses to differing step heights and step cadences, in young and middle-aged females.

	Middle-Aged				Young			
				Step Cadence (steps·min^-1^)				
Step Height (cm)	*80*	*90*	*100*	*110*	*80*	*90*	*100*	*110*
	*Heart Rate (beats·min*^*-1*^)*							
**17**	116.4 ± 14.74	121.7 ± 15.75	126.8 ± 15.73	134.9 ± 14.89	115.2 ± 20.72	121.6 ± 23.13	124.3 ± 22.03	130.5 ± 23.83
**25**	126.8 ± 12.26	135.0 ± 13.37	141.9 ± 15.28	149.4 ± 15.51	128.5 ± 22.70	134.0 ± 22.86	137.6± 22.12	146.5 ± 21.09
**30**	138.0 ± 16.38	147.9 ± 15.95	152.4 ± 15.69	161.8 ± 14.70	136.4 ± 22.89	146.5 ± 25.04	155.1 ± 24.73	159.9 ± 23.04
**34**	147.6 ± 13.92	154.6 ± 14.34	160.1 ± 10.60	166.8 ± 9.21	148.5 ± 21.32	151.1 ± 20.87	159.3 ± 22.31	166.7 ± 20.45
	Heart rate reserve (%)*†							
**17**	59.7 ± 14.04	65.1 ± 14.63	69.9 ± 15.53	78.4 ± 14.12	45.0 ± 14.32	50.2 ± 16.53	52.2 ± 15.67	57.2 ± 17.34
**25**	70.1 ± 11.58	78.0 ± 12.92	85.5 ± 13.83	93.0 ± 14.19	54.9 ± 16.73	59.9 ± 16.82	62.9 ± 15.96	69.9 ± 15.44
**30**	80.5 ± 15.40	90.9 ± 15.56	96.05 ± 14.85	105.7 ± 13.52	62.2 ± 16.65	70.3 ± 18.20	74.8 ± 18.07	80.7 ± 17.23
**34**	91.2 ± 13.76	98.5 ± 14.75	104.0 ± 10.86	110.6 ± 8.80	72.1 ± 16.04	73.6 ± 14.75	79.9 ± 16.61	86.0 ± 15.61
	*Metabolic Cost (Kcal·min*^*-1*^)*							
**17**	5.25 ± 0.81	5.66 ± 0.95	6.15 ± 1.01	6.76 ± 1.13	5.13 ± 0.86	5.70 ± 0.87	6.08 ± 0.96	6.63 ± 1.03
**25**	6.14 ± 0.90	6.87 ± 0.94	7.43 ± 0.90	8.01 ± 0.94	6.21 ± 1.06	6.81 ± 0.97	7.23 ± 1.04	7.93 ± 1.12
**30**	7.32 ± 1.12	7.88 ± 1.18	8.20 ± 1.09	8.70 ± 1.25	7.08 ± 1.02	7.90 ± 1.16	8.43 ± 1.12	9.00 ± 1.28
**34**	7.95 ± 1.23	8.36 ± 0.98	8.93 ± 0.93	9.43 ± 1.14	8.01 ± 1.03	8.75 ± 1.25	9.35 ± 1.3	10.16 ± 1.44

*Within-group differences are significant (p<0.01).

*†Between-group* differences are significant (p>0.01).

Values are means ± SD.

There was a significant third order interaction effect (step height*step cadence*age group) for METs (*P* = 0.02, η_p_^2^ = .094) indicating a slight non-linear response. As evident in Fig 2, for the middle-aged group linear trends begin to converge at higher step heights and rates, showing a slight plateau in the metabolic response. Significant interaction effects for HR (step height*step cadence: *P* = 0.046, η^2^ = .080) and %HRr (step height*step cadence: P = 0.043, η_p_^2^ = .081; step cadence*group: *P*<0.001, η_p_^2^ = .351) and metabolic cost (step height*group: P = 0.036, η_p_^2^ = .103) were also detected.

### Multilevel Regression Analysis

A model for estimating the MET intensity of stepping exercise was developed from step height, step cadence and age (Eq 1). The percentage of additional variance accounted for by the inclusion of leg length (-0.066 ± 0.086) was not significant. Similarly, the addition of stature (-0.087 ± 0.045) to the model did not account for additional variance in METs and did not significantly alter the percentage variance explained by the initial three predictors. The estimated parameters from the multilevel regression analysis including the three fixed factors and the two sources of random variation between- and within-individuals are given in Table 3.

METs=(a+b⋅step height)+(c⋅step cadence)+(d⋅age) Eq 1

**Table 3:** Multilevel regression analysis of METs, using step height, step cadence, and age as predictor variables.

	Fixed Explanatory Variables	Value
	Constant (*a*)	-3.743 ± 0.695
	Step Height (*b*)	0.188 ± 0.004
	Step Cadence (*c*)	0.060 ± 0.002
	Age (*d*)	0.053 ± 0.015
	Variance-Covariance Matrix of Random Variables	
		Constant (*a*)
		*Level 1 (within individuals)*
	Constant (*a*)	0.332 ± 0.023
		*Level 2 (between individuals)*
	Constant (*a*)	1.731 ± 0.468
Values are means ± SE		

## Discussion

The aim of the present study was to describe the physiological responses to bench stepping exercise in young and middle-aged females in order to develop an equation that will allow the prescription of step exercise (using step height and step cadence) at a target intensity known to enhance health and fitness in a given population. It was found that METs, HR and metabolic cost vary considerably in response to differing step heights and cadences, which supports previous research in the area of aerobic dance bench stepping [33, 36, 37]. Our results show that both step height and step cadence have a significant independent effect on the physiological responses to stepping exercise. In both the young and middle-aged groups, each 10 beat.min^-1^ increase in step cadence resulted in a 0.6-MET increase in intensity, while each mean increase of 6 cm in step height resulted in a 1-MET increase in intensity. We further extend this line of research by reporting that the acute responses to stepping at different heights and cadences vary across different age groups. Results revealed that at a given step height and cadence, the older females were required to work at an intensity on average 1.9 ± 0.26 METs greater than the younger females. For example, a step height and cadence combination that corresponded to an intensity of 9 METs (75%HRr) in the younger group resulted in 10.8 METs (96% HRr) in the middle-aged group, an intensity that is close to maximal exertion. Since ageing is accompanied by a decline in the efficiency of the cardiovascular system [38] and in skeletal muscle quantity [39], these results are not entirely unexpected. At a step height of 30 cm, the middle-aged steppers are already operating close to their maximum capability suggesting that further increases in step height are unnecessary and impractical for this age group. Furthermore, these age-related differences provide additional evidence that previously published equations for step exercise [40] may significantly underestimate the metabolic cost of this activity in older populations, resulting in inappropriate exercise prescriptions. Our results, therefore, lend credence to the importance of tailored exercise programmes for individuals of different ages.

Stepping exercise has the potential to be promoted on a public health level as a scalable HIIT model, as it is low-cost, simple to perform, and may be particularly appealing to those who have restricted access to exercise facilities or little time to exercise. With this, as well as the need for personalised prescriptions in mind, a further aim of our study was to develop a useful means of prescribing intensity-specific stepping exercise to female adults of different ages. By choosing a step height that is readily accessible to the individual, it is possible to calculate the step cadence required to reach a desired age-adjusted vigorous intensity level (Eq 1). As previously mentioned, stair climbing exercise is associated with a number of short and long-term positive health outcomes [1–10], and so replicating the physiological responses of this everyday activity through bench stepping exercise should result in similar benefits. It is therefore recommended that users of the prescription equation select an intensity of 8.8 METs (equivalent to stair climbing) in order to prescribe an appropriate stepping cadence based on their age and the step height available to them. Furthermore, to reflect a scaled HIIT protocol, stepping bouts of 3 minutes, interspersed with up to 3 minutes of recovery, are suggested. Personalised exercise prescription such as this may improve adherence to exercise programmes in inactive populations [41]. By addressing a number of common barriers to exercise participation using stepping as a vehicle for scalable HIIT, there is a greater likelihood of longer term adherence to the exercise. Furthermore, high intensity exercise has been linked with heightened mood post exercise [42] and higher levels of enjoyment compared with moderate intensity exercise [43, 44]. For example, Bartlett and co-workers [43] have shown higher ratings of perceived enjoyment following 3 minute bouts of HIIT compared with 50 minutes of continuous exercise. Further examination of training responses, as well as comparison with traditional exercise programmes and more contemporary HIIT protocols, is required in order to elucidate the long term benefits of this scalable exercise prescription. Those wishing to use this equation to prescribe exercise training to individuals should consider the principle of overload to ensure continued cardiorespiratory fitness adaptations as training progresses.

By including an accurate measurement of RMR into the calculation of MET intensity, the quality of the prediction for the individual work performed by participants has been improved. This measurement also allowed comparison of resting metabolism between the age groups, and with the standard resting VO_2_ value of 3.5ml·kg^-1^·min^-1^ [20], which is still commonly cited in research. Our results show that young and older females have a 12% and 33% lower RMR than the standard value, respectively, further highlighting the inadequacy of this standardisation in certain populations [21, 45]. It should be noted that the RMR measurement used in this study did not account for differences in lean body mass between groups, which may have further refined our results. Byrne and colleagues [21] have shown BMI to be strongly correlated with fat mass in a heterogeneous sample. Therefore, it is possible that the slightly higher average BMI in our middle-aged group could have reflected a higher fat mass and contributed to the lower RMR values compared with our younger group. Importantly, this study introduces a more accurate representation of the relative MET intensity of stepping exercise across two different age groups.

### Limitations

The study sample was restricted to healthy Caucasian females, within two age ranges, and therefore the reported results are not truly representative of the general population. Lean muscle mass was not measured in the present study, data from which may have helped to explain age-related difference in RMR. Further, due to impracticalities it was not possible to complete all testing on the same day. While every effort was made to present participants for testing in the same physiological state, day-to-day variability in may have introduced some error to calculations.

### Conclusions

The ability to prescribe stepping exercise at a specific intensity that replicates stair climbing means that the health benefits of the latter can potentially be promoted to a larger population. This study confirms that stepping can replicate the physiological demands of stair climbing in both a young and middle-aged female population, however younger females require higher step heights and faster cadences compared with their middle-aged counterparts, to reach higher levels of exercise intensity. The importance of innovative exercise methods to improve health and fitness in a practical, time-efficient manner across various populations is growing. This study provides a simple means of prescribing an intensity-specific step exercise programme, which may be performed in small amounts throughout the day, as a scalable form of HIIT, to females of different ages.

## Supporting Information

S1 Dataset Study data. (SAV)
